# Prognostic and therapeutic roles of B7-H3 and B7-H4 in prostate cancer

**DOI:** 10.1007/s12672-026-04926-0

**Published:** 2026-04-19

**Authors:** Yunxi Hu, Wenjiang Yang, Shuang Guo, Denghui Huang

**Affiliations:** 1 Wenshang County People’s Hospital, 1 Dehui Road, Jining, 272500 China; 2 Urological Surgical Department, Wenshang County People’s Hospital, 1 Dehui Road, Jining, 272500 Shandong China

**Keywords:** Prostate cancer, B7-H3, CD276, B7-H4, VTCN1, Immune checkpoint, Immunotherapy, Antibody–drug conjugate, CAR T-cell, Prognostic biomarker, Tumor microenvironment, Castration-resistant prostate cancer

## Abstract

Prostate cancer (PCa) remains a leading cause of cancer-related morbidity and mortality in men worldwide, with metastatic castration-resistant prostate cancer (mCRPC) presenting a particularly formidable therapeutic challenge. The limited efficacy of conventional immunotherapies targeting the PD-1/PD-L1 axis in PCa has spurred intensive research into alternative immune checkpoints. Among the B7 family of immunomodulatory proteins, B7-H3 (CD276) and B7-H4 (VTCN1/B7x/B7S1) have emerged as critical regulators of PCa pathogenesis, progression, and therapeutic resistance. This comprehensive review synthesizes current evidence from preclinical and clinical studies to delineate the multifaceted roles of B7-H3 and B7-H4 in PCa. We first detail the expression patterns of these molecules in PCa tissues and their robust correlation with adverse clinicopathological features, including high Gleason score, advanced tumor stage, metastasis, and poor survival outcomes. We then explore the complex molecular mechanisms underlying their pro-tumorigenic functions, encompassing immune suppression (inhibition of T-cell activity, modulation of myeloid-derived suppressor cells [MDSCs] and tumor-associated macrophages [TAMs]) and non-immune effects (regulation of cancer stem cells [CSCs], DNA damage repair [DDR], androgen receptor [AR] signaling, and tumor dormancy). Furthermore, we systematically review the rapidly expanding landscape of therapeutic strategies targeting B7-H3 and B7-H4, including antibody–drug conjugates (ADCs), chimeric antigen receptor (CAR) T-cell therapy, monoclonal antibodies, bispecific agents, and combination therapies with radiotherapy, AR pathway inhibitors (ARPIs), or other immunotherapies. We also discuss emerging directions such as liquid biopsy for B7-H3 detection, B7-H4-targeted immunoPET imaging, racial disparities in B7-H3 expression, and the development of predictive biomarkers for treatment response. Collectively, this review establishes B7-H3 and B7-H4 as pivotal nodes in PCa biology and highlights their promising potential to improve the clinical management of advanced and treatment-resistant PCa through improved prognostic stratification and the development of novel precision therapies.

## Introduction

Prostate cancer (PCa) is the second most commonly diagnosed cancer and a leading cause of cancer-related death among men globally, with an estimated 1.4 million new cases and 375,000 deaths annually [1, 2]. The clinical spectrum of PCa is highly heterogeneous, ranging from indolent localized disease manageable with active surveillance to aggressive metastatic disease that progresses to castration-resistant prostate cancer (CRPC) [3]. Androgen deprivation therapy (ADT) is the cornerstone of treatment for advanced PCa, but nearly all patients eventually develop CRPC, a lethal stage characterized by resistance to hormonal therapies and limited therapeutic options [4].

The advent of immunotherapy has revolutionized the treatment of numerous malignancies, but its success in PCa has been modest. Checkpoint inhibitors targeting the PD-1/PD-L1 axis have shown limited efficacy in unselected PCa populations, attributed to the disease’s characteristically low mutational burden, sparse T-cell infiltration, and profoundly immunosuppressive tumor microenvironment (TME) [5, 6]. This therapeutic gap has intensified the search for alternative immune regulatory pathways that could serve as prognostic biomarkers and therapeutic targets.

The B7 family of cell-surface proteins, which includes both co-stimulatory and co-inhibitory ligands, plays a central role in fine-tuning T-cell responses and maintaining immune homeostasis [7]. Beyond the well-characterized PD-1/PD-L1 and CTLA-4 pathways, newer members of this family, particularly B7-H3 and B7-H4, have garnered significant attention due to their frequent overexpression in PCa and other solid tumors, combined with their restricted expression in normal tissues, which minimizes the risk of on-target, off-tumor toxicity [8, 9].

B7-H3, initially identified as a potential co-stimulatory molecule for T-cell activation[10], has since been predominantly characterized as a potent negative regulator of T-cell function, inhibiting proliferation, cytokine production, and cytolytic activity [11, 12]. Its overexpression in PCa has been consistently linked to aggressive disease features and poor clinical outcomes [13, 14]. In contrast, B7-H4 is a well-established negative regulator of T-cell immunity [15, 16], and its expression in PCa is associated with tumor grade and immunosuppressive TME remodeling, particularly through its association with tumor-associated macrophages (TAMs) and regulation of tumor dormancy [17, 18].

Over the past decade, significant advances have been made in understanding the molecular mechanisms by which B7-H3 and B7-H4 drive PCa progression, their regulation by key oncogenic pathways (for example, AR signaling, DDR pathways, PTEN/TP53 loss), and their potential as therapeutic targets. This review aims to consolidate these findings into a comprehensive framework, highlighting the latest preclinical and clinical developments and identifying critical gaps that require further investigation. By integrating insights from molecular biology, translational research, and clinical trials, we provide a roadmap for the future integration of B7-H3 and B7-H4 targeting into personalized PCa treatment strategies.

For this narrative review, we conducted a comprehensive literature search using the PubMed, Web of Science. The search covered the period from January 2000 to December 2025. Keywords and MeSH terms included “prostate cancer,” “B7-H3,” “CD276,” “B7-H4,” “VTCN1,” “immune checkpoint,” “immunotherapy,” “prognosis,” “tumor microenvironment,” and “clinical trial.” We focused on peer-reviewed original research articles, review articles, and registered clinical trials relevant to the prognostic and therapeutic roles of B7-H3 and B7-H4 in prostate cancer. Priority was given to studies with large cohorts, mechanistic depth, and direct translational relevance.

### B7-H3 in prostate cancer: expression, prognostic significance, and clinical correlates

#### Tissue expression patterns of B7-H3 in PCa

B7-H3 is a type I transmembrane glycoprotein that exhibits distinct expression patterns in PCa compared to benign prostate tissue. Multiple immunohistochemical (IHC) studies have consistently demonstrated that B7-H3 is highly overexpressed in PCa tissues, with expression levels significantly exceeding those in normal or benign prostatic epithelium [13, 14, 19]. A landmark study analyzed a large cohort of PCa patient biopsies and reported that membranous B7-H3 (mB7-H3) is expressed in 97% of hormone-sensitive prostate cancer (HSPC) samples and 93% of CRPC samples, indicating that B7-H3 upregulation is an early event in the natural history of aggressive PCa [13]. This high prevalence across disease stages makes B7-H3 a particularly attractive target for both early and advanced PCa.

B7-H3 expression is primarily localized to tumor epithelial cells, with some expression observed on endothelial cells within the TME [13]. Notably, B7-H3 exhibits significant intra-tumoral heterogeneity, but even with this variability, high expression levels have been consistently associated with adverse pathological features [13, 14]. In a large-scale analysis of over 17,000 PCa specimens, study found that 47% of tumors showed positive B7-H3 staining (12.3% weak, 21.1% moderate, 13.5% strong), with no detectable expression in normal prostatic glands [19]. This tumor-specific expression profile minimizes the risk of on-target, off-tumor toxicity, a critical advantage for therapeutic development.

Longitudinal studies have shown that B7-H3 expression remains relatively stable as PCa progresses from HSPC to CRPC, with infrequent conversion from positive to negative or vice versa [13]. This stability is a valuable characteristic for biomarker development, as archival HSPC tissue samples can reliably inform treatment decisions in the CRPC setting. Additionally, B7-H3 expression is maintained in aggressive PCa subtypes, including neuroendocrine prostate cancer (NEPC) and RB1-deficient CRPC, further expanding its therapeutic relevance [20, 21].

#### Prognostic significance of B7-H3

The prognostic value of B7-H3 in PCa has been robustly validated across multiple independent cohorts. High B7-H3 expression is consistently associated with a range of adverse clinical outcomes, including high Gleason score, advanced pathological stage, extraprostatic extension, seminal vesicle invasion, lymph node metastasis, biochemical recurrence (BCR), and PCa-specific mortality [13, 14, 19, 22].

Roth et al. (2007) were among the first to establish the prognostic significance of B7-H3, reporting that marked B7-H3 intensity (present in 19.8% of PCa cases) was associated with a fourfold increased risk of cancer progression after radical prostatectomy, even after adjusting for established prognostic factors [14]. Zang et al. (2007) confirmed these findings in a cohort of 823 patients, demonstrating that strong B7-H3 expression correlated with disease spread and poor survival, with a hazard ratio (HR) of 3.48 for cancer-specific death [22]. More recently, Amori et al. (2021) evaluated B7-H3 expression in diagnostic biopsy specimens from metastatic PCa patients and found that high B7-H3 expression (≥ 50% of tumor cells with moderate-strong staining) was an independent predictor of worse 5-year disease-specific survival (64% vs. 86%) and overall survival (40% vs. 62%) [23].

Notably, the prognostic impact of B7-H3 appears to be context-dependent, with varying significance across molecular subtypes. Bonk et al. (2020) reported that B7-H3 retains its independent prognostic value particularly in ERG-negative cancers, where it serves as a strong risk factor for BCR [19]. This finding underscores the importance of integrating B7-H3 expression with molecular subtype information for optimal risk stratification. Additionally, B7-H3 expression has been linked to treatment resistance, with high levels correlating with reduced sensitivity to ADT, radiotherapy, and chemotherapy [24, 25].

#### Racial and ancestral disparities in B7-H3 expression

Emerging evidence highlights significant racial and ancestral differences in B7-H3 expression, which may contribute to the well-documented disparities in PCa incidence and outcomes. Mendes et al. (2022) conducted a landmark study across racially diverse cohorts and found that B7-H3 protein expression was significantly lower in tumors from men of African ancestry compared to those of European ancestry [26]. In a grade-matched cohort, the average B7-H3 H-score was nearly 40% lower in self-identified Black patients, and this association was robustly validated by genetic ancestry markers (r = − 0.42 for African ancestry, r = 0.44 for European ancestry) [26]. Importantly, this disparity was independent of known molecular drivers such as ERG fusion status and PTEN deletion, suggesting inherent differences in tumor immunobiology across ancestral groups [26]. The lower B7-H3 expression in men of African ancestry raises critical questions about the utility of B7-H3-targeted therapies in this high-risk population and implies the existence of alternative immune evasion mechanisms that warrant further investigation. These findings emphasize the need for mandatory inclusion of racially and ancestrally diverse cohorts in future translational research and clinical trials to ensure equitable advancements in PCa care.

### B7-H3 in liquid biopsy: a non-invasive biomarker

The development of non-invasive liquid biopsy approaches for B7-H3 has emerged as a promising tool for real-time disease monitoring, particularly in metastatic PCa where repeated tissue biopsies are challenging. Ju et al. (2025) recently developed a novel “B7-H3 + EV Assay” that uses click chemistry to enrich B7-H3-positive extracellular vesicles (EVs) from patient plasma, followed by RT-qPCR quantification of a reference gene (ACTB) to generate a B7-H3 + EV score [27].

This assay demonstrated significant clinical utility: B7-H3 + EV scores were significantly higher in mCRPC patients compared to metastatic hormone-sensitive prostate cancer (mHSPC) patients or healthy donors, increased with disease progression from mHSPC to mCRPC, and served as an independent predictor of shorter overall survival (HR = 2.19) [27]. Importantly, longitudinal monitoring showed that B7-H3 + EV scores dynamically reflected treatment response and disease progression, at times correlating more accurately with clinical status than serum prostate-specific antigen (PSA) [27]. This liquid biopsy platform provides a rapid, reliable, and cost-effective method for monitoring B7-H3 expression, positioning it as an ideal companion diagnostic for B7-H3-targeted therapies.

## Molecular mechanisms of B7-H3 in prostate cancer progression

**Fig. 1:**
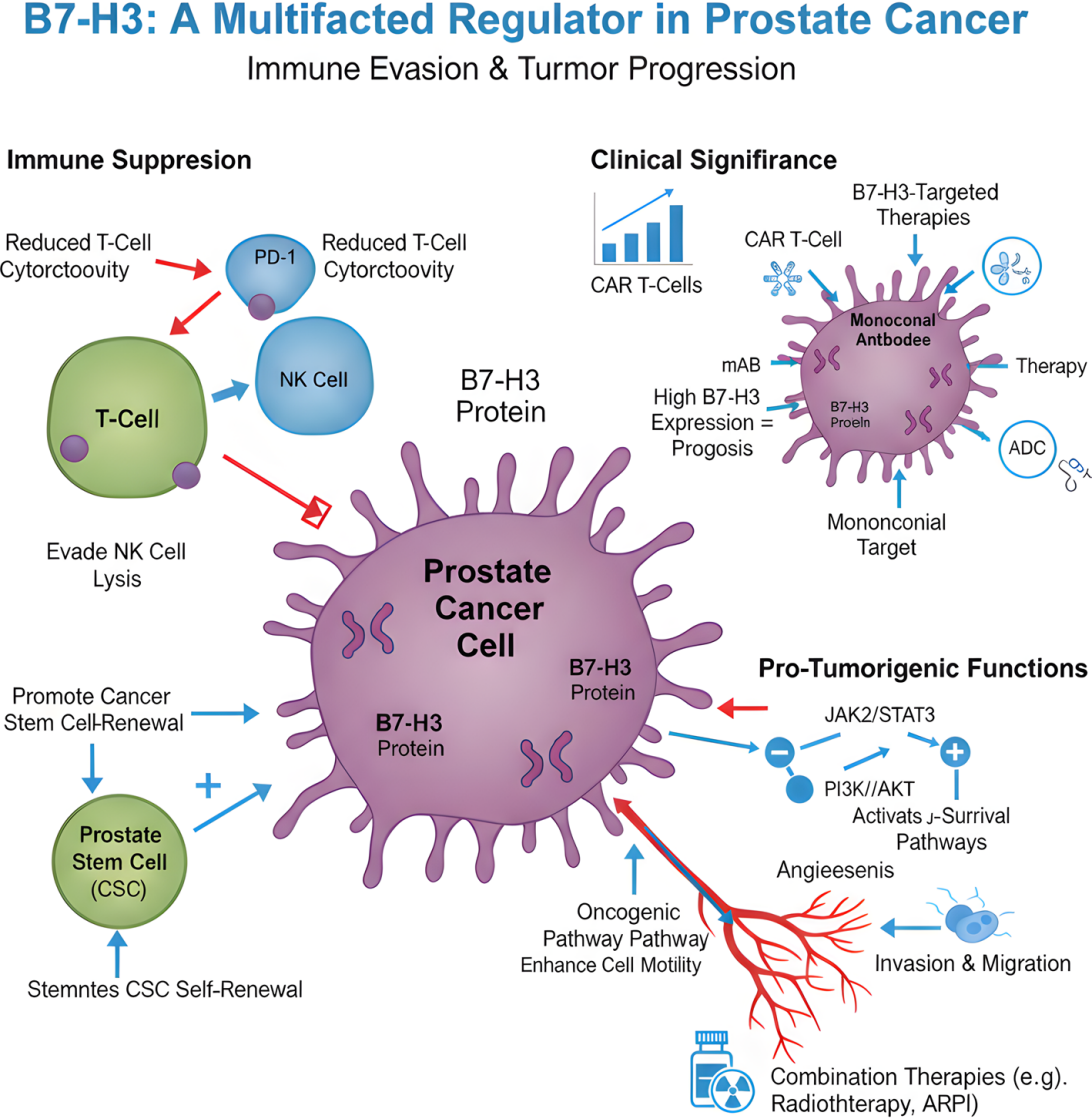
B7-H3 as a Multifaceted Regulator in Prostate Cancer: Immune Evasion and Tumor Progression. illustrates the diverse biological roles of B7-H3, highlighting its contribution to immune evasion, tumor progression, and cancer stem cell renewal in prostate cancer. The diagram shows that B7-H3 expression on prostate cancer cells suppresses anti-tumor immune responses by reducing T-cell cytotoxicity, interacting with PD-1, and enabling cancer cells to evade NK-cell–mediated lysis. Clinically, high B7-H3 expression correlates with poor prognosis, and has become an emerging target for monoclonal antibodies, antibody–drug conjugates (ADCs), and CAR T-cell therapies. On the tumor-intrinsic side, B7-H3 activates oncogenic signaling pathways including JAK2/STAT3 and PI3K/AKT, promoting cancer cell survival, proliferation, invasion, and migration. The diagram also highlights B7-H3-mediated angiogenesis, which enhances nutrient and oxygen supply to the tumor. Additionally, B7-H3 supports cancer stem cell (CSC) maintenance and self-renewal, further facilitating tumor progression. Combination therapeutic strategies, such as radiotherapy and androgen-receptor pathway inhibitors (ARPI), are shown as potential complementary approaches to B7-H3-targeted treatments

B7-H3 contributes to PCa progression through a complex interplay of immune-dependent and immune-independent mechanisms, integrating with key oncogenic pathways to drive tumor growth, metastasis, and therapeutic resistance (Fig. 1).

### Immune-suppressive functions in the tumor microenvironment

The primary immune-regulatory role of B7-H3 in PCa is to foster an immunosuppressive TME, enabling tumor cells to evade anti-tumor immunity. Multiple studies have demonstrated that B7-H3 inhibits T-cell function by suppressing proliferation, cytokine production (e.g., IFN-γ, IL-2), and cytolytic activity [11, 12, 28]. Guo et al. (2023) showed that CRPC biopsies with high mB7-H3 expression had significantly lower densities of intra-tumoral CD3 + T-cells, indicating a role in excluding or suppressing T-cell infiltration [13].

Beyond T-cell inhibition, B7-H3 modulates other immune cell populations within the TME. Zhou et al. (2020) uncovered a novel mechanism by which B7-H3 promotes PCa progression in mouse models: B7-H3 directly antagonizes myeloid-derived suppressor cell (MDSC)apoptosis both in vitro and in vivo, sustaining this immunosuppressive cell population [29]. Depletion of immune cells with cyclophosphamide abrogated the growth advantage of B7-H3-overexpressing tumors, confirming the immune-dependent nature of this mechanism [29]. B7-H3 has also been shown to impair natural killer (NK) cell function by promoting the binding of MHC-I molecules to inhibitory receptors on NK cells, further facilitating immune escape [30].

### Non-immune pro-tumorigenic functions

In addition to its immune-regulatory roles, B7-H3 exerts direct effects on tumor cells and cancer stem cells (CSCs), contributing to PCa progression through T-cell-independent mechanisms. Zhang et al. (2021) reported that B7-H3 is naturally expressed at higher levels on prostate cancer stem cells (PCSCs; ALDH + CD44 +) than on bulk tumor cells in human PCa cell lines (DU145, PC3) [25]. Fractionated irradiation (FIR), a standard PCa treatment, significantly upregulates B7-H3 expression on both bulk and stem cells, a finding that directly links B7-H3 to the radioresistance phenotype of CSCs [25].

B7-H3 also promotes tumor cell migration, invasion, and epithelial-to-mesenchymal transition (EMT) through the activation of key signaling pathways such as JAK2-STAT3, PI3K-AKT, and NF-κB [31, 32]. Liu et al. (2015) demonstrated that B7-H3 enhances cell migration and invasion in colorectal cancer through the JAK2/STAT3/MMP9 pathway, a mechanism likely conserved in PCa [31]. Furthermore, B7-H3 reprograms glucose metabolism towards aerobic glycolysis (the Warburg effect) by stabilizing HIF1α through reactive oxygen species (ROS)-mediated mechanisms, providing tumor cells with the metabolic flexibility to survive under hypoxic conditions [33].

### Limitations of current evidence and conflicting findings

While the pro-tumorigenic role of B7-H3 is well-established, a critical appraisal of the literature reveals several limitations and areas of conflicting evidence. First, the function of B7-H3 appears context-dependent. Although predominantly an inhibitor, initial studies identified it as a T-cell co-stimulator [10], and some models suggest it can have host-protective roles, the determinants of which remain unclear. Second, its prognostic value, while robust overall, may vary by molecular subtype; for instance, its independent prognostic power is particularly strong in ERG-negative cancers [19], suggesting interaction with other oncogenic drivers. Third, much of the mechanistic data, particularly for non-immune functions like metabolic reprogramming [33], relies heavily on murine models and established cell lines, raising questions about reproducibility and translation to the heterogeneous human disease. Finally, the risk of immune checkpoint redundancy is high; targeting B7-H3 alone may lead to compensatory upregulation of other inhibitory pathways like PD-L1 or B7-H4, necessitating combination strategies.

## Regulation by key oncogenic pathways

### Androgen receptor (AR) signaling

The relationship between B7-H3 and AR signaling is complex and context-dependent. Multiple studies have shown a positive correlation between B7-H3 expression and AR activity, with B7-H3 highly expressed in AR-positive PCa cell lines and associated with AR mRNA levels and an AR activity signature in patient tumors [13, 17]. Benzon et al. (2017) identified a functional androgen response element upstream of the B7-H3 gene, and chromatin immunoprecipitation (ChIP) assays confirmed direct AR binding to the B7-H3 promoter [17].

Intriguingly, the regulatory effect of AR on B7-H3 appears to be bidirectional. In LNCaP cell lines, the presence of androgens (dihydrotestosterone, DHT) suppresses B7-H3 expression, indicating that AR signaling can act as a transcriptional repressor [17]. This negative regulation has profound clinical implications: ADT-induced suppression of AR signaling may lift this repression, leading to transient upregulation of B7-H3, a phenomenon observed in the early phases of ADT [34]. As tumors progress to CRPC, B7-H3 expression is reactivated, often surpassing pre-ADT levels, due to AR reactivation or alternative oncogenic pathways [34]. This dynamic regulation suggests that the timing of B7-H3-targeted therapies relative to ADT may be critical for optimizing efficacy.

### PTEN/TP53 loss and Sp1-mediated transcription

Recent work has identified the loss of tumor suppressor genes PTEN and TP53 as key drivers of B7-H3 overexpression in aggressive PCa. Shi et al. (2023) demonstrated that B7-H3 is the most significantly overexpressed immune checkpoint in PCa tumors harboring co-inactivating alterations in PTEN and TP53, a genomic event enriched in over 60% of CRPCs [35]. This relationship was validated across multiple models, including The Cancer Genome Atlas (TCGA) data, single-cell RNA-seq, genetically engineered mouse models (GEMMs), and human cell lines [35].

The molecular mechanism linking PTEN/TP53 loss to B7-H3 upregulation involves the transcription factor Sp1. ChIP assays confirmed that Sp1 directly binds to the promoter region of the CD276 gene, and luciferase reporter assays demonstrated that Sp1 knockdown suppresses CD276 promoter activity [35]. The PTEN-PI3K-AKT axis activates Sp1, while p53 and Sp1 engage in a negative feedback loop where depletion of p53 augments Sp1 and B7-H3 expression [35]. Importantly, Sp1-mediated B7-H3 upregulation is only unleashed upon the concurrent inactivation of both PTEN and p53, highlighting the specificity of this regulatory pathway in aggressive PCa [35].

### Epigenetic regulation and DDR deficiencies

Epigenetic mechanisms also play a critical role in regulating B7-H3 expression in PCa. Yamada et al. (2023) demonstrated that DNA methyltransferases (DNMTs), particularly DNMT1, are highly upregulated in NEPC and RB1-deficient CRPC [20]. Genetic knockout or pharmacological inhibition of DNMTs (e.g., with decitabine) resulted in hypomethylation of the B7-H3 promoter and significant upregulation of B7-H3 expression [20]. This finding has therapeutic implications, as DNMT inhibition can be used to “prime” B7-H3-low tumors by increasing target expression, thereby sensitizing them to B7-H3-directed therapies [20].

B7-H3 expression is also strongly associated with DNA damage repair (DDR)deficiencies. Guo et al. (2023) reported that B7-H3 levels are significantly higher in CRPC tumors with deleterious alterations in DDR genes, particularly biallelic loss of BRCA2 and ATM [13]. This association links B7-H3 to genomic instability, a feature known to modulate the immune landscape in other cancers, and provides a rationale for combining B7-H3-targeted therapies with PARP inhibitors or DNA-damaging agents [13].

## Therapeutic strategies targeting B7-H3 in prostate cancer

The consistent overexpression of B7-H3 in PCa, its association with aggressive disease, and its multifaceted pro-tumorigenic functions make it a premier candidate for targeted therapy. A diverse array of therapeutic modalities is under development, ranging from monoclonal antibodies to cell-based therapies, with promising preclinical and early clinical results.

### Antibody–drug conjugates (ADCs)

ADCs represent a powerful class of targeted therapeutics that deliver a potent cytotoxic payload directly to antigen-expressing cells, minimizing off-target toxicity. Several B7-H3-targeted ADCs are currently in preclinical or clinical development for PCa.

#### DS-7300a (ilmatamab deruxtecan)

DS-7300a is a B7-H3-targeting ADC conjugated to a topoisomerase I inhibitor payload (DXd). Guo et al. (2023) evaluated DS-7300a in a comprehensive panel of PCa models, demonstrating potent, dose-dependent antitumor activity in vitro against B7-H3-positive PCa cell lines and patient-derived organoids (PDX-Os) of both adenocarcinoma and neuroendocrine histology [13]. In vivo, DS-7300a induced significant tumor growth inhibition and complete regressions in B7-H3-positive patient-derived xenograft (PDX) models [13]. Pharmacodynamic analyses confirmed on-target effects, including reduced proliferation (Ki67) and induction of senescence markers (p21, SA-β-gal) in treated tumors [13]. A phase I/II clinical trial (NCT04145622) is currently evaluating DS-7300a in advanced solid tumors, including PCa, with preliminary reports showing a promising safety profile and efficacy [36].

#### MGC018 (vobramitamab duocarmazine)

MGC018 is an ADC consisting of a humanized anti-B7-H3 monoclonal antibody conjugated to duocarmycin, a DNA alkylating agent. Preclinical studies have demonstrated potent antitumor activity in PCa models, with the ADC effectively targeting both cancer cells and tumor-associated vasculature [37]. Early clinical results from a phase I cohort expansion study (NCT03729596) showed that MGC018 induces significant PSA declines in a subset of patients with metastatic CRPC [38]. A phase II trial (NCT05555117) is ongoing to further evaluate its efficacy in advanced PCa.

#### Epigenetic priming for ADC therapy

The epigenetic regulation of B7-H3 has led to the development of combination strategies involving DNMT inhibitors and B7-H3-targeted ADCs. Yamada et al. (2023) demonstrated that in B7-H3-low PCa models, combination therapy with decitabine (a pan-DNMT inhibitor) and DS-7300a resulted in enhanced B7-H3 expression and a synergistic antitumor response [20]. This “epigenetic priming” strategy is particularly promising for treating aggressive, RB1-deficient CRPC and NEPC, where DNMT overexpression is common [20].

### Chimeric antigen receptor (CAR) T-cell therapy

CAR T-cell therapy involves genetically engineering a patient’s T-cells to recognize and kill tumor cells expressing a specific antigen. B7-H3 is an ideal target for CAR T-cell therapy due to its high and frequent expression on PCa cells and limited expression on normal tissues.

Zhang et al. (2021) developed B7-H3-directed CAR T-cells and tested them against radioresistant PCa [25]. Their findings were striking: B7-H3 CAR T-cells were more effective at killing PCSCs than bulk tumor cells (correlating with higher basal B7-H3 expression on CSCs), FIR pre-treatment enhanced PCSC susceptibility to CAR T-cell killing by upregulating B7-H3 expression, and the combination of FIR and B7-H3 CAR T-cells was significantly more effective than either modality alone in CRPC mouse models [25]. The optimal antitumor effect was achieved when CAR T-cells were administered 1–3 days post-FIR, coinciding with the peak of FIR-induced B7-H3 upregulation [25].

Li et al. (2023) constructed a second-generation B7-H3 CAR using a humanized scFv derived from the 8H9 antibody, with CD28 and CD3ζ signaling domains [39]. These CAR T-cells demonstrated potent and specific anti-tumor activity in vitro, inducing near-complete elimination of B7-H3-positive PCa cells and releasing high levels of effector cytokines (IFN-γ, TNF-α) [39]. In vivo, a single infusion of B7-H3 CAR T-cells significantly suppressed tumor growth in immunodeficient mice bearing DU145 xenografts, without causing observable toxicity [39]. Several early-phase clinical trials are underway (NCT04691713, NCT04432649) to evaluate the safety and efficacy of B7-H3-targeted CAR T-cells in solid tumors, including PCa [36].

### Monoclonal antibodies

Monoclonal antibodies targeting B7-H3 aim to block its immunosuppressive functions and/or induce antibody-dependent cellular cytotoxicity (ADCC) against B7-H3-expressing tumor cells.

Enoblituzumab (MGA271) is an Fc-optimized humanized anti-B7-H3 monoclonal antibody designed to enhance ADCC. A phase II neoadjuvant trial (NCT02923180) in men with localized high-risk PCa showed that enoblituzumab was well-tolerated and induced a favorable decline in PSA [40]. More importantly, it triggered significant immune activation within the TME, including increased T-cell and myeloid cell infiltration [40]. An interim analysis of a phase I trial (NCT02381314) evaluating enoblituzumab in combination with ipilimumab (anti-CTLA-4) showed promising antitumor activity in patients with treatment-refractory PCa [41].

Despite these promising results, limitations such as variable single-agent efficacy, the need for predictive biomarkers to identify responders, and potential immune-related adverse events from combination therapies require further investigation.

### Bispecific antibodies and T-cell engagers

Bispecific antibodies and T-cell engagers (BiTEs) are designed to simultaneously bind to a tumor antigen (e.g., B7-H3) and a T-cell surface molecule (e.g., CD3), redirecting T-cell cytotoxicity to tumor cells. Obrinolatamab (MGD009) is a humanized bispecific DART molecule that binds to B7-H3 on tumor cells and CD3 on T-cells. Preclinical studies have shown potent antitumor activity in PCa cell lines [42].

The clinical translation of bispecific agents faces challenges, including cytokine release syndrome and on-target, off-tumor toxicity, which necessitate careful dose optimization and patient monitoring.

### Combination therapeutic strategies

Given the complex nature of PCa pathogenesis and therapeutic resistance, combination strategies involving B7-H3-targeted agents are being actively explored to enhance efficacy. Rational combinations include:

With Radiotherapy: As demonstrated by Zhang et al. (2021), radiotherapy upregulates B7-H3 expression, creating a window of opportunity for enhanced CAR T-cell or ADC activity [25]. This combination also targets radioresistant CSCs, a key driver of recurrence.

With AR Pathway Inhibitors: The link between B7-H3 and AR signaling suggests potential synergy. Combining B7-H3 inhibitors with androgen receptor pathway inhibitors (ARPIs) like abiraterone or enzalutamide could simultaneously cripple a key oncogenic driver and an associated immune evasion mechanism [35].

With DDR Inhibitors: Given the association between B7-H3 and DDR deficiencies (e.g., BRCA2/ATM loss), combinations with PARP inhibitors may be particularly effective in this subset of PCa [13].

With Other Immunotherapies: Dual checkpoint blockade (e.g., B7-H3 + PD-L1 or CTLA-4 inhibitors) has shown synergistic effects in preclinical models. Shi et al. (2023) reported that combining B7-H3 and CTLA-4 inhibition with enzalutamide cured 76.5% of mice with CRPC, establishing durable immunological memory [35].

## B7-H4 in prostate cancer: expression, functional roles, and therapeutic potential

While research on B7-H4 in PCa is less extensive than for B7-H3, emerging evidence positions B7-H4 as a critical regulator of PCa progression, particularly in the context of tumor dormancy and immunosuppressive TME remodeling.

### Expression and prognostic significance of B7-H4

Like B7-H3, B7-H4 is frequently overexpressed in PCa tissues compared to normal prostate epithelium. Qian et al. (2010) provided foundational evidence showing that B7-H4 is diffusely expressed in the cytoplasm and/or membrane of prostate carcinoma tissues, with expression levels significantly higher than in normal prostate tissues [43]. Critically, B7-H4 expression was significantly elevated in patients with higher clinical tumor grades (II–III) compared to those with lower-grade (I) disease [43].

Li et al. (2020) confirmed these findings, demonstrating that B7-H4 is significantly upregulated in PCa tissues compared to benign prostatic hyperplasia (BPH) [44]. In PCa, B7-H4 is diffusely expressed in the cytoplasm of luminal epithelial cells, a shift from its restricted expression in hyperplastic basal cells in BPH [44]. Positive B7-H4 expression was significantly correlated with higher pathological T-stage (pT3–4) and advanced clinical stage, and Kaplan–Meier survival analysis revealed that PCa patients with high B7-H4 expression had significantly shorter overall survival [44]. Multivariate Cox regression analysis confirmed B7-H4 as an independent poor prognostic factor, alongside lymph node metastasis [44].

### Functional roles of B7-H4

#### Immune suppression and TAM association

B7-H4 functions primarily as a negative regulator of T-cell immunity, inhibiting T-cell proliferation, cell cycle progression, and the development of effector functions [15, 16]. Its overexpression in PCa is hypothesized to help tumor cells evade immune surveillance by dampening anti-tumor T-cell responses [43].

A key emerging role of B7-H4 is its association with tumor-associated macrophages (TAMs). Kumar et al. (2025) developed a B7-H4-specific immunoPET radiotracer ([89Zr]Zr-DFO-2H9) and demonstrated that the dominant source of B7-H4 expression in immunocompetent PCa models is not tumor cells but infiltrating TAMs, particularly M2-like (CD206 +) TAMs [17]. Depletion of macrophages with clodronate liposomes significantly reduced the B7-H4-specific PET signal, confirming this association [17]. This finding positions B7-H4 as a valuable non-invasive biomarker for visualizing the immunosuppressive TAM compartment and monitoring response to macrophage-targeted therapies [17].

#### Regulation of tumor dormancy

Perhaps the most novel function of B7-H4 in PCa is its role in maintaining tumor dormancy following ADT. Kang et al. (2025) demonstrated that B7-H4 is selectively upregulated during the dormant phase induced by ADT, with expression peaking 3 months post-castration and declining upon relapse to CRPC [18]. This upregulation is specific to the dormant state and is inversely correlated with proliferation markers (Ki67) and serum PSA levels [18].

The induction of B7-H4 appears to be a direct response to AR suppression, as B7-H4 upregulation occurs after both surgical castration and pharmacological AR blockade (e.g., enzalutamide) but not after chemotherapy [18]. Single-cell RNA sequencing of dormant PDX models revealed that B7-H4-positive cells are enriched in pathways related to ECM-receptor interaction and regulation of the actin cytoskeleton [18]. Functional studies showed that forced B7-H4 expression in LNCaP cells reduces proliferation under androgen-deficient conditions and delays tumor relapse in castrated mice, suggesting that B7-H4 facilitates the interaction of dormant PCa cells with the remodeled ECM to promote quiescence [18].

#### Link to cancer stemness

Li et al. (2020) uncovered a novel association between B7-H4 and cancer stemness in PCa [44]. B7-H4 expression was positively correlated with the expression of stemness-related genes, including SOX2, SOX9, and CD44, in human PCa tissues [44]. Knocking down B7-H4 in PCa cell lines (PC3 and DU145) led to downregulation of these stemness markers, impaired tumorsphere formation (an in vitro measure of self-renewal), and reduced migratory and invasive capacities [44]. B7-H4 also drives proliferation and survival through the PI3K/Akt signaling pathway, as its expression correlates with increased pPI3K and pAkt levels [44].

Despite these insights, the precise signaling receptor for B7-H4 and its downstream effectors in regulating stemness remain largely unknown, representing a key area for future research.

### Diagnostic and therapeutic potential of B7-H4

#### B7-H4-targeted therapies

Building on the success of B7-H3 ADCs, B7-H4 is now being pursued as a therapeutic target. AZD8205, a B7-H4-targeted ADC conjugated to a topoisomerase I inhibitor, is the most advanced agent in this class and is currently in a Phase I/II clinical trial for advanced solid tumors [45]. Its mechanism of action could involve direct killing of B7-H4-positive tumor cells, but given the prominent expression of B7-H4 on immunosuppressive TAMs [17], AZD8205 may also exert its effect by selectively depleting this pro-tumorigenic immune population. This dual targeting of the tumor and its supportive microenvironment is a particularly attractive feature. Other modalities, such as bispecific antibodies (e.g., B7-H4xCD3 T-cell engagers) and CAR T-cells, are in preclinical development and hold promise for targeting B7-H4-expressing cells, including the dormant tumor cell population [18].

#### ImmunoPET imaging

The association of B7-H4 with TAMs has led to the development of B7-H4-targeted immunoPET imaging as a diagnostic tool. The [89Zr]Zr-DFO-2H9 tracer developed by Kumar et al. (2025) enables non-invasive assessment of the immunosuppressive TAM compartment, providing a powerful tool for patient stratification and monitoring response to TAM-targeted therapies [17]. This tracer could identify patients with “immune-hot but suppressive” tumors who might benefit from TAM-depleting or TAM-reprogramming therapies [17].

#### Targeted therapeutic strategies

While B7-H4-targeted therapies are less advanced than those for B7-H3, the foundational evidence supports their development. The approaches used for B7-H3, such as ADCs and CAR T-cells, are directly applicable to B7-H4. AZD8205, a B7-H4-targeted ADC, is currently in clinical development for solid tumors and could selectively eliminate B7-H4-positive TAMs, potentially converting “cold” tumors into “hot” ones and synergizing with T-cell-directed immunotherapies [45].

**Fig. 2:**
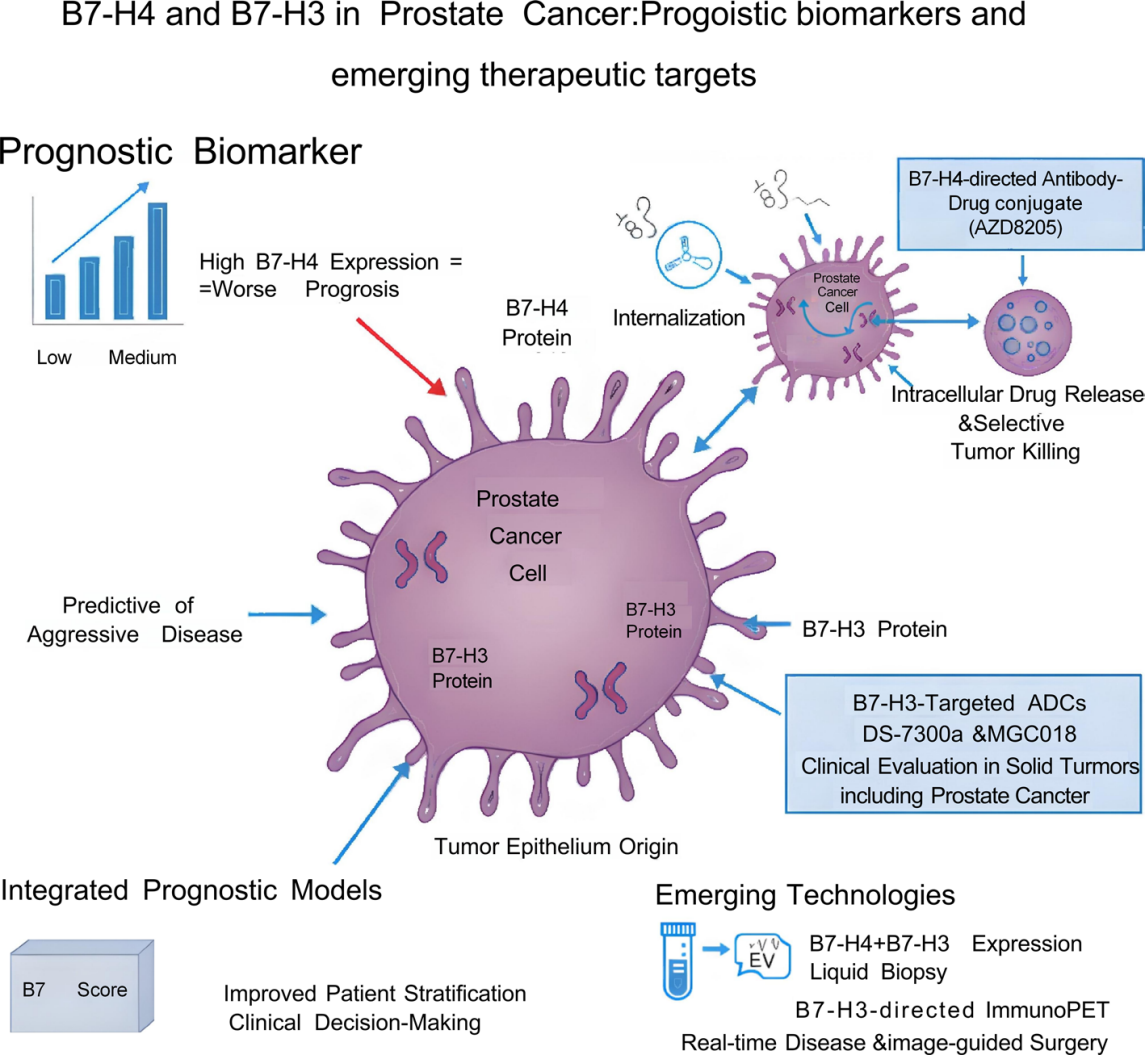
B7-H4 and B7-H3 in prostate cancer: prognostic biomarkers and emerging therapeutic targets. This schematic summarizes how B7-H4 and B7-H3 contribute to prostate cancer biology, risk stratification, and therapeutic development. Left: Increasing B7-H4 expression from low to high is associated with poorer clinical outcome, and the illustrated prostate cancer cell is annotated as “predictive of aggressive disease” to emphasize the value of B7-H4 as a prognostic biomarker. Integration of B7-H4 levels into composite prognostic algorithms (e.g., “B7 score”) is depicted as refining patient stratification and supporting clinical decision-making. Right: A B7-H4-directed antibody–drug conjugate (ADC), AZD8205, binds B7-H4 on the surface of prostate cancer cells, undergoes internalization, and releases a cytotoxic topoisomerase I inhibitor payload intracellularly, thereby mediating selective tumor-cell killing. Representative B7-H3-targeted ADCs under clinical evaluation in solid tumors, such as DS-7300a and MGC018, are indicated as parallel approaches that exploit B7-H3 expression on prostate cancer cells. Center: A stylized prostate cancer cell is shown to express both B7-H4 and B7-H3, highlighting their biological origin within the tumor epithelium. Bottom: Emerging technologies include liquid biopsy–based assessment of B7-H4/B7-H3 expression and B7-H3-directed ImmunoPET imaging to enable real-time disease monitoring and image-guided surgery. Collectively, the figure illustrates the roles of B7-H4 and B7-H3 as clinically actionable prognostic biomarkers and therapeutic targets in prostate cancer

B7-H4 also presents a unique opportunity to target minimal residual disease during the dormant phase and prevent CRPC relapse. Therapeutic strategies could include B7-H4-directed ADCC antibodies, bispecific antibodies (B7-H4xCD3), or CAR T-cells to actively kill B7-H4-positive dormant cells [18]. Alternatively, antibodies that modulate B7-H4 signaling could prolong the dormant state indefinitely, effectively turning PCa into a chronic, manageable disease (Fig. 2) [18].

**Fig. 3:**
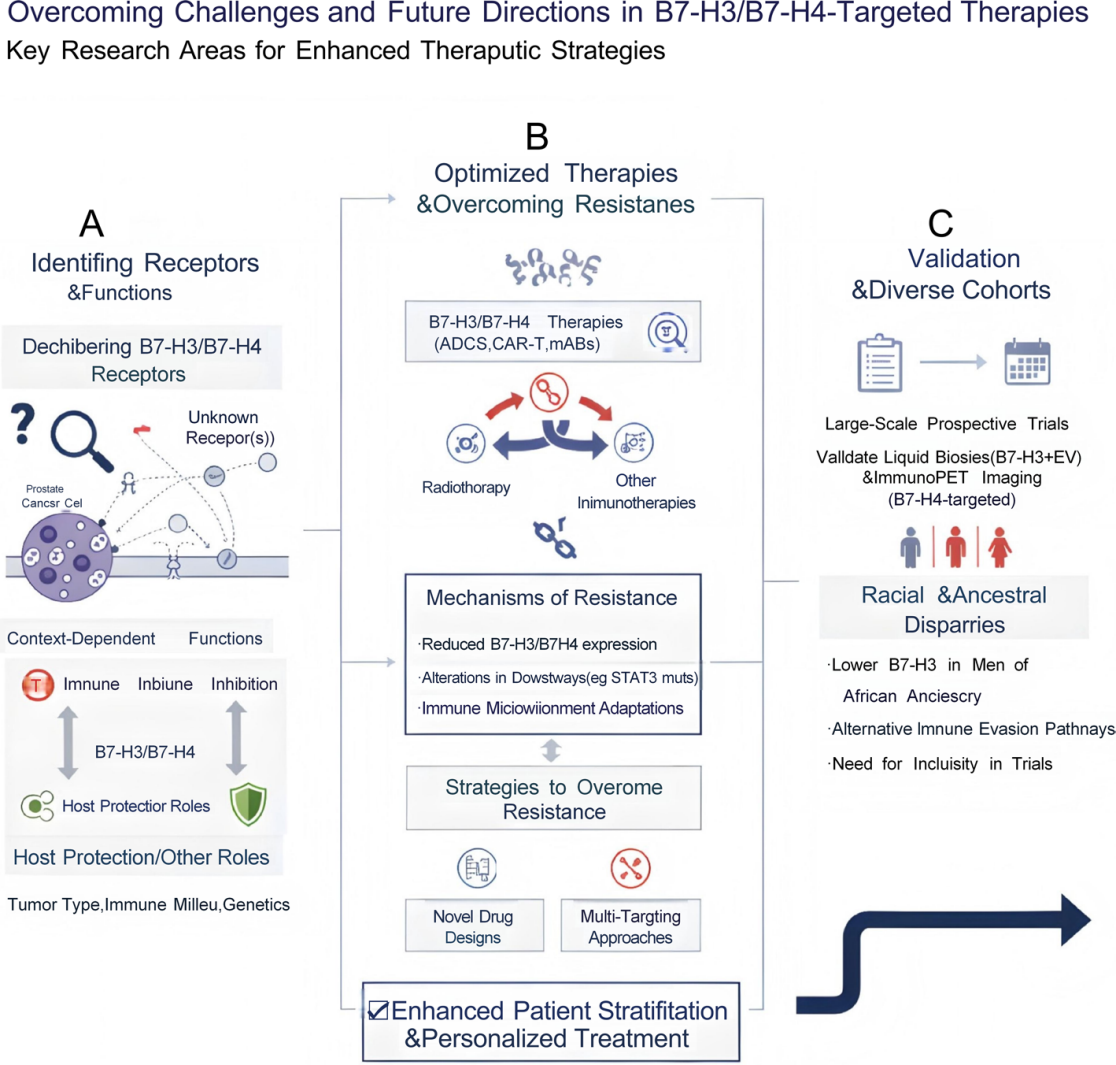
Overcoming Challenges and Future Directions in B7-H3/B7-H4-Targeted Therapies. This schematic summarizes key research priorities and translational barriers in the development of B7-H3/B7-H4-directed cancer therapies. **A** Identifying receptors and functional roles: The figure highlights the incomplete characterization of B7-H3/B7-H4 receptors, noting the presence of currently unknown binding partners. Their immunological functions are depicted as context-dependent, ranging from immune inhibition to host-protective responses, and influenced by tumor type, immune milieu, and genetic background. **B** Optimizing therapies and overcoming resistance: Current therapeutic platforms, including antibody–drug conjugates (ADCs), monoclonal antibodies, and CAR T-cells, are shown alongside mechanisms of therapeutic resistance, such as reduced B7-H3/B7-H4 expression, alterations in downstream signaling pathways, and adaptive remodeling of the immune microenvironment. The figure further illustrates strategies to overcome resistance, such as novel drug designs and multi-targeting approaches, and acknowledges bidirectional interactions with immunotherapy and radiotherapy. **C** Validation and inclusion of diverse cohorts: The need for rigorous clinical validation of liquid biopsy and immunoPET imaging approaches for B7-H3/B7-H4 assessment is emphasized. The panel also notes racial and ancestral differences in B7-H3 expression, specifically lower levels observed in men of African ancestry, and underscores the importance of diverse clinical trial representation and evaluation of alternative immune-evasion pathways. Collectively, the illustration emphasizes that resolving these challenges will support improved patient stratification and the development of personalized therapeutic strategies targeting B7-H3 and B7-H4

A significant challenge for B7-H4-directed therapy is its predominant expression on TAMs rather than tumor cells in some contexts, which may complicate direct tumor cell killing and require strategies focused on modulating the TME (Fig. 3).

## Integrated perspectives and emerging directions

### Comparative overview of B7-H3 and B7-H4 in prostate cancer

To provide a clear conceptual framework, Table 1 summarizes the key similarities and differences between B7-H3 and B7-H4 in the context of PCa. Both molecules function as immune checkpoints, are overexpressed in tumors, correlate with poor prognosis, and are being explored as therapeutic targets. However, they exhibit distinct non-immune functions: B7-H3 is more strongly linked to cancer stem cell maintenance, radioresistance, and direct promotion of tumor cell motility, whereas B7-H4 is uniquely associated with regulating tumor dormancy post-ADT and is a prominent marker of immunosuppressive TAMs. These differences suggest complementary roles in PCa progression and highlight the potential for combination strategies targeting both pathways to achieve more comprehensive anti-tumor effects (Table 2).

**Table 1:** Comparative overview of B7-H3 and B7-H4 in prostate cancer

Feature	B7-H3 (CD276)	B7-H4 (VTCN1/B7x/B7S1)
Expression in PCa	Highly overexpressed in tumor epithelium; stable from HSPC to CRPC	Overexpressed in tumor epithelium and strongly associated with TAMs
Prognostic value	Strong independent predictor of high grade, stage, metastasis, and poor survival	Independent predictor of advanced stage and shorter overall survival
Key immune functions	Inhibits T-cell and NK-cell activity; sustains MDSCs	Inhibits T-cell activation; major expression on immunosuppressive M2-like TAMs
Key non-immune functions	Promotes CSC phenotype, radioresistance, migration/invasion, metabolic reprogramming	Regulates tumor dormancy post-ADT; linked to cancer stemness and ECM interaction
Therapeutic modalities in development	ADCs (DS-7300a, MGC018), CAR T-cells, monoclonal antibodies (enoblituzumab), bispecific agents	ADC (AZD8205), immunoPET imaging ([89Zr]Zr-DFO-2H9), potential for CAR T-cells
Major challenges	Racial disparities in expression; unknown receptor; context-dependent functions	Predominant expression on TAMs; unknown receptor; role in dormancy needs therapeutic exploitation

**Table 2:** Clinical Development Landscape of B7-H3 and B7-H4 Targeted Therapies in Prostate Cancer

Target	Agent (company/partner)	Class	Phase	Key trial identifier (s)	Relevant prostate cancer data/status
B7-H3	Ifinatamab Deruxtecan (I-DXd, DS-7300) (Daiichi Sankyo/Merck)	ADC (topo I inhibitor)	III	CTR20252497 (China, mCRPC)	Global frontrunner B7-H3 ADC; phase III initiated in mCRPC
B7-H3	BNT324 (DB-1311) (DualityBio/BioNTech)	ADC (topo I inhibitor)	III	NCT06672987	Reported ORR 42.3% in mCRPC patients; phase III ongoing
B7-H3	HS-20093 (GSK5764227) (Hansoh Pharma/GSK)	ADC (topo I inhibitor)	III	NCT07099898 (ES-SCLC)	BTD in SCLC; exploring expansion into prostate cancer
B7-H3	YL201 (YL Biologics/AstraZeneca)	ADC (topo I inhibitor)	III	NCT06612151, NCT06629597	Data published in *Nature Medicine*; prostate cancer trials anticipated
B7-H3	MHB088C (Minghui Pharma/Qilu Pharma)	ADC (topo I inhibitor)	III	Not disclosed	Fourth B7-H3 ADC in phase III; prostate cancer potential
B7-H3	B7-H3 CAR-T (CARsgen, Legend Biotech, etc.)	CAR T-cell	I	NCT04483778, NCT04691713	Early-phase trials in prostate cancer and other solid tumors
B7-H4	AZD8205 (AstraZeneca)	ADC (topo I inhibitor)	I/II	NCT05123482	Fastest B7-H4 ADC; dose expansion underway; prostate cancer cohort may be included
B7-H3/PD-L1	DB-1419 (DualityBio)	Bispecific ADC	I	IND approved (China)	Novel bispecific ADC; potential for prostate cancer

*ADC* antibody–drug conjugate, *BTD* Breakthrough Therapy Designation, *ES-SCLC* extensive-stage small cell lung cancer, *mCRPC* metastatic castration-resistant prostate cancer, *ORR* objective response rate, *topo I* topoisomerase I

### The B7 score and immune phenotyping

Recognizing that PCa often expresses multiple B7 family members, Zhou et al. (2021) proposed a “B7 score” that integrates the expression of B7-H3 and HHLA2 (another B7 family member) [46]. They found that high expression of either B7-H3 or HHLA2 is associated with aggressive clinicopathological features and poor survival, and the B7 score is a more powerful independent prognostic factor than either molecule alone [46]. When combined with CD8 + T-cell density, the B7 score can stratify patients into four immune subtypes:

Immune Type I (Low B7, High CD8): Most favorable prognosis.

Immune Type II (Low B7, Low CD8):Immunologically ignorant.

Immune Type III (High B7, High CD8): Potential for adaptive immune resistance, where checkpoint blockade may be most effective.

Immune Type IV (High B7, Low CD8): “Super-cold” tumors, most profoundly immunosuppressive and lethal [46].

This classification provides a refined framework for prognostic prediction and therapy selection, guiding the use of B7-H3/B7-H4-targeted therapies in patients most likely to benefit.

### Molecular imaging for surgical guidance

Beyond therapeutic targeting, B7-H3 is emerging as a valuable target for improving surgical precision. Tian et al. (2024) developed a B7-H3-targeted NIR-II fluorescence molecular imaging probe (AbB7-H3-800CW) that can specifically visualize PCa tumors in mouse models with high contrast [47]. This probe offers superior resolution and deeper tissue penetration compared to traditional NIR-I imaging, enabling precise delineation of tumor margins during surgery and detection of occult lesions as small as 1 mm [47]. Ex vivo validation with fresh human PCa tissues confirmed that cancer tissues exhibit significantly higher fluorescence intensity than normal tissues, correlating with B7-H3 expression levels [47]. This technology has the potential to reduce positive surgical margins and improve outcomes in radical prostatectomy.

### Optimizing combination therapies

The future of B7-H3/B7-H4-targeted therapy lies in rational combinations that address the complex biology of PCa. Key considerations for combination therapy design include:

Timing of ADT and B7-H3 Targeting: The transient upregulation of B7-H3 following ADT suggests that combining B7-H3-targeted agents with ADT may be most effective in the early phases of treatment [34].

Epigenetic Priming: For B7-H3-low tumors, combining DNMT inhibitors with B7-H3 ADCs can enhance target expression and improve efficacy [20].

Dual Checkpoint Blockade: Combining B7-H3 inhibition with PD-L1 or CTLA-4 blockade addresses compensatory immune suppression and improves response rates [35].

Targeting CSCs and TAMs: Combinations that target B7-H3-positive CSCs (e.g., CAR T-cells + radiotherapy) and B7-H4-positive TAMs (AZD8205 + PD-1 inhibitors) may overcome multiple layers of therapeutic resistance.

### Limitations of current evidence and future research priorities

Despite significant progress, several critical questions and limitations remain. First, the definitive receptors for B7-H3 and B7-H4 remain unknown, which fundamentally limits our ability to understand their intrinsic signaling and develop effective blocking antibodies. The success of ADCs and CAR T-cells, which rely on target binding rather than receptor blockade, circumvents this issue but does not resolve the biological ambiguity. Second, the field is limited by a lack of critical appraisal. Many studies, including those cited here, are preclinical with small sample sizes and may not fully account for context-dependent functions or the risk of immune redundancy. Third, there is an over-reliance on murine models that may not faithfully recapitulate the human TME or the complexities of the immune system. Fourth, the mechanisms of resistance to B7-H3/B7-H4-targeted therapies are unknown and require proactive investigation. Finally, a significant gap is the lack of diversity in study cohorts. The finding of lower B7-H3 expression in men of African ancestry [26] underscores this point and demands that future research and clinical trials prioritize inclusion of racially and ancestrally diverse populations to ensure equitable benefits.

## Conclusion

B7-H3 and B7-H4 have emerged as pivotal regulators of PCa pathogenesis, progression, and therapeutic resistance, with distinct yet complementary roles. Substantial evidence supports that B7-H3 is a robust prognostic biomarker and therapeutic target, characterized by its high overexpression in PCa, association with aggressive disease features, and multifaceted pro-tumorigenic functions (immune suppression, promotion of CSCs, integration with key oncogenic pathways). The development of B7-H3-targeted therapies, including ADCs, CAR T-cells, and monoclonal antibodies, has shown promising preclinical and early clinical efficacy, particularly in combination with radiotherapy, AR pathway inhibitors, or other immunotherapies.

B7-H4, while less extensively studied, appears to play critical roles in tumor dormancy, TAM modulation, and cancer stemness, positioning it as a valuable diagnostic biomarker and potential therapeutic target for preventing CRPC relapse. B7-H4-targeted immunoPET imaging offers non-invasive assessment of the immunosuppressive TME, while B7-H4-directed therapies hold promise for eliminating dormant tumor cells.

The integration of B7-H3 and B7-H4 into prognostic models (the B7 score) and immune phenotyping frameworks provides a refined approach to patient stratification, ensuring that targeted therapies are delivered to those most likely to benefit. Emerging technologies such as liquid biopsies and molecular imaging further enhance the clinical utility of these molecules, enabling real-time disease monitoring and improved surgical precision.

Despite these advances, several challenges remain, including the identification of B7-H3/B7-H4 receptors, the elucidation of context-dependent functions, and the optimization of combination therapies to overcome resistance. Future research should focus on addressing these gaps and validating novel therapeutic strategies in large-scale prospective clinical trials, particularly in racially and ancestrally diverse cohorts. Importantly, current knowledge is limited by a lack of diversity in study cohorts and the need for large-scale prospective validation of novel diagnostic and therapeutic tools.

In conclusion, targeting the B7-H3 and B7-H4 pathways represents a paradigm shift in PCa treatment, offering new hope for patients with advanced, treatment-resistant disease. By leveraging the multifaceted roles of these molecules as prognostic biomarkers, diagnostic tools, and therapeutic targets, we can work towards improving the clinical management of PCa and ultimately reduce mortality from this devastating disease.

## Data Availability

No datasets were generated or analysed during the current study.
